# Osteogenic and Antibacterial Response of Levofloxacin-Loaded Mesoporous Nanoparticles Functionalized with *N*-Acetylcysteine

**DOI:** 10.3390/pharmaceutics17040519

**Published:** 2025-04-15

**Authors:** Alberto Polo-Montalvo, Natividad Gómez-Cerezo, Mónica Cicuéndez, Blanca González, Isabel Izquierdo-Barba, Daniel Arcos

**Affiliations:** 1 Departamento Química en Ciencias Farmacéuticas, Facultad de Farmacia, Universidad Complutense de Madrid, Plaza Ramón y Cajal s/n, 28040 Madrid, Spain; albpolo@ucm.es (A.P.-M.); magome21@ucm.es (N.G.-C.); mcicuendez@ucm.es (M.C.); blancaortiz@ucm.es (B.G.); 2 Instituto de Investigación Sanitaria del Hospital Clínico San Carlos (IdISSC), 28040 Madrid, Spain; 3 Instituto de Investigación Sanitaria, Hospital 12 de Octubre i+12, 28040 Madrid, Spain; 4 CIBER de Bioingeniería, Biomateriales y Nanomedicina—CIBER-BBN, 28040 Madrid, Spain

**Keywords:** mesoporous bioactive nanoparticles, *N*-acetylcysteine, levofloxacin, bone infection, bone regeneration

## Abstract

**Background/Objectives**: Bone infection is one of the most prevalent complications in orthopedic surgery. This pathology is mostly due to bacterial pathogens, among which *S. aureus* stands out. The formation of a bacterial biofilm makes systemic treatment with antibiotics ineffective. Herein we propose a nanosystem composed of mesoporous bioactive glass nanoparticles (MBGN) loaded with levofloxacin and functionalized with N-acetylcysteine (NAC), aiming to offer an alternative to current treatments. These nanoparticles would present antibacterial activity able to disintegrate the biofilm and regenerate the peri-implantar osseous tissue. **Methods**: MBGN of composition 82.5 SiO_2_—17.5 CaO have been synthesized, loaded with levofloxacin, and functionalized with NAC (MBGN-L-NAC). The antimicrobial activity against mature *S. aureus* biofilms and bioactivity of the nanosystem have been evaluated, as well as its biocompatibility and ability to promote murine pre-osteoblastic MC3T3-E1 differentiation. **Results**: MBGNs exhibited high surface areas and radial mesoporosity, allowing up to 23.1% (% *w/w*) of levofloxacin loading. NAC was covalently bound keeping the mucolytic thiol group, SH, available. NAC and levofloxacin combination enhances the activity against *S. aureus* by disrupting mature biofilm integrity. This nanosystem was biocompatible with pre-osteoblasts, enhanced their differentiation towards a mature osteoblast phenotype, and promoted bio-mimetic mineralization under in vitro conditions. MBGN-L-NAC nanoparticles induced greater osteogenic response of osteoprogenitor cells through increased alkaline phosphatase expression, increased mineralization, and stimulation of pre-osteoblast nodule formation. **Conclusions**: MBGN-L-NAC exhibits a more efficient antibacterial activity due to the biofilm disaggregation exerted by NAC, which also contributes to enhance the osteoinductive properties of MBGNs, providing a potential alternative to conventional strategies for the management of bone infections.

## 1. Introduction

Osteomyelitis is a serious and often painful bone infection. It can occur in any bone in the body but is most common in the long bones of the arms and legs, as well as the spine and pelvis. Osteomyelitis associated with bone prosthesis implant is one of the most frequent infectious complications in traumatology. Its incidence is around 5%, and gram-positive microorganisms are generally isolated [1]. Periprosthetic bone infection (PJI) can be caused by many pathogens, the most common being Gram-positive bacteria such as *S. epidermidis* and *S. aureus*. These bacteria form a biofilm composed of extracellular polymeric substances (EPS), which shields them from antibiotics and the immune system. The biofilm also facilitates infection spread by releasing colony-forming units [2,3]. The biofilm is composed of proteins and polysaccharides stabilized by strong and weak unions as Van der Waals forces, hydrogen bonds, and disulfide bonds [3,4].

Bone infections are often treated with high doses of antibiotics, such as levofloxacin, an antibiotic that targets the topoisomerase complex of Gram-positive and Gram-negative bacteria [5]. However, once the pathogens have developed a biofilm, they become highly resistant to antibiotics [6], and it becomes necessary to remove the prosthesis and debride the tissue to avoid chronic osteomyelitis or even patient death by septicemia. On the other hand, the osteolysis caused in the peri-implant tissues leads to loosening of the prostheses, requiring their replacement even after the infection has been eradicated [1].

Mesoporous bioactive glass nanoparticles (MBGNs) are nanomaterials with bone-repairing properties [7] that have been used as drug and ions-delivery systems, due to their high surface area and tunable chemical composition [8]. The therapeutic properties of MBGNs can be enhanced by doping with therapeutic ions such as manganese and zinc to improve the osteogenic properties of MBGNs [9,10], and copper and silver to impart antibacterial properties to MBGNs [11,12]. MBGNs can also be chemically modified by adding functional groups including amine-functionalization for modification of surface charge [13,14,15], drug loading [16], and functionalization with polyethyleneimine for miRNA loading and delivery [17]. Their high surface area and mesoporous structure allow them to be loaded with different drugs for osteogenic [18], antitumoral [19], antibiotic [20], or antiosteoporotic purposes [21], which have been shown to be non-toxic and excretable [22]. Moreover, multifunctional MBGNs nanoplatforms with stimuli-responsive properties and theranostic applications have been recently engineered following strategies such as surface functionalization, incorporation of other nanostructures, or modification of the network [23].

*N*-acetylcysteine (NAC) is an amino acid widely used in clinical practice [24]. First used as an antidote for acetaminophen intoxication, NAC has a major application in the treatment of mucus-producing respiratory infections, as its sulfhydryl group can break the disulfide bonds that bind the mucopolysaccharide matrix that forms mucus, dissolving it [25] and preventing EPS production [26]. NAC has been used alone or in combination with antibiotics to eliminate mature biofilms [27,28,29,30] and plays a key role in regulating the intracellular redox state as an exogenous supply of cysteine, the direct precursor of the redox-regulating molecule, glutathione [31,32]. Reactive oxygen species (ROS) and redox state play a key role in the immune activity [33] and by extension, in infection control and recovery, where ROS scavenging helps regenerate tissue damaged by the infection [34].

In this study, we have developed a nanosystem based on MBGNs to provide an alternative to the current treatment of bone defects caused by *S. aureus* infections. The prepared MBGNs are loaded with levofloxacin and subsequently functionalized with NAC to take advantage of its mucolytic properties. This combination is expected to help the nanosystem to disaggregate mature *S. aureus* biofilms and locally deliver the levofloxacin content. Certainly, there is a variety of antibiofilms agents that include enzymes, peptides, antibiotics, polyphenols, etc., which follow different mechanisms of action such as inhibition of AHL-mediated quorum-sensing pathway, cleavage of peptidoglycan, dispersion of extracellular polymeric substance (EPS) of biofilm, etc. The choice of NAC responds to the multifunctional nature of our nanosystem. In this sense, the contribution of NAC is not limited to disintegrate the EPS of the biofilm, but it provides immunomodulatory activity of inflammation and osteogenic activity to enhance the bioactive process induced by MBGNs.

Since MBGNs exhibit osteogenic properties and antibiotic efficacy against biofilm-forming bacteria, we anticipate that this nanosystem may become an adjuvant to the treatment of bone infection, including PJI. In this last scenario, the treatment of infected intraosseous prostheses usually involves removal and replacement of the prosthesis, especially when biofilm forms on the implant surface. Local antibiotic delivery systems, such as gentamicin-loaded PMMA beads, are limited to acting as prophylaxis systems, once the prosthesis has been replaced. The nanosystem proposed in this work has been designed to locally treat bone infections that have developed bacterial biofilm, having antibacterial efficacy in clinical scenarios where most antibiotics fail. We envision that these nanosystems could provide a device capable of eliminating the infection in intraosseous implants and regenerating the peri-implant bone, avoiding loosening of the prosthesis and its subsequent replacement.

## 2. Materials and Methods

### 2.1. Synthetic Procedure

MBGNs were synthesized following Xie et al. protocol [35] to obtain a radial pore structure. Briefly, using an oil-in-water biphasic stratification reaction, 12 g of hexadecyltrimethylammonium bromide (CTAB, ABCR, Karlsruhe, Germany) and 0.36 mL of triethanolamine (TEA, Sigma-Aldrich, St. Louis, MO, USA) were dissolved in 108 mL of distilled water for 1 h at 150 rpm and 60 °C. Then, a solution of 12 mL of tetraethyl orthosilicate (TEOS, Sigma-Aldrich, St. Louis, MO, USA), 0.912 mL of triethyl phosphate (TEP, Sigma-Aldrich, St. Louis, MO, USA) and 48 mL of cyclohexane (Panreac, Barcelona, Spain) was then gently added to form an oil-in-water biphasic system and was stirred for 24 h at 150 rpm and 60 °C. After discarding the oleic phase by aspiration, the aqueous phase was centrifuged, and the pellet was washed three times with 30 mL of pure ethanol and dried at 30 °C under vacuum to obtain the nanoparticles. A total of 1 g of nanoparticles were suspended in 40 mL of pure ethanol containing 0.704 g of Ca(NO_3_)_2_·4H_2_O (Sigma-Aldrich, St. Louis, MO, USA) to obtain a nominal composition of 80 SiO_2_—4 P_2_O_5_—16 CaO (% mol). The suspension was stirred at room temperature (RT) until complete evaporation of the ethanol. The pellet was gently ground and then calcined at 600 °C for 5 h under air atmosphere to obtain the mesoporous bioactive glass nanoparticles (MBGN) as a disaggregated fine white powder.

### 2.2. Antibiotic Loading

300 mg of MBGN were suspended in 60 mL of a 16 mM levofloxacin (Sigma-Aldrich) solution in dichloromethane (Panreac, Barcelona, Spain) for 24 h at 300 rpm at RT in darkness. This concentration involves a highly saturated levofloxacin solution previously used for this drug impregnation on porous materials [36]. The resulting levofloxacin-loaded nanoparticles (MBGN-L) were washed twice with 30 mL of pure ethanol and dried at 30 °C under vacuum for 24 h.

### 2.3. N-Acetylcysteine (NAC) Anchoring

**Scheme 1:**
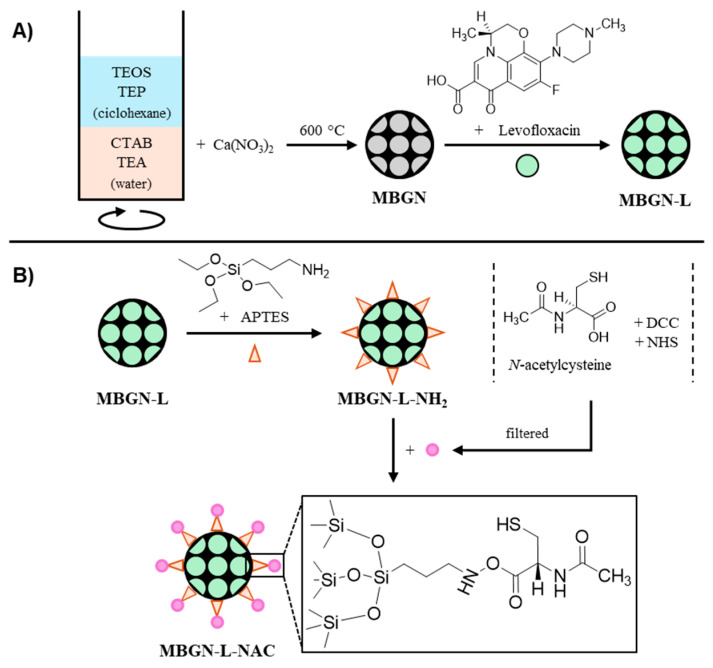
Schematic representation of the synthesis of the different nanomaterials prepared in this work. (**A**) Synthesis of MBGN and levofloxacin loading to obtain MBGN-L. (**B**) Functionalization of MBGN-L with APTES for NAC anchoring to obtain MBGN-L-NAC.

With the purpose of anchoring NAC with its mucolytic thiol group exposed, 300 mg of MBGN-L were suspended in 30 mL of anhydrous toluene (Thermo Scientific, Waltham, MA, USA) under N_2_ atmosphere. Then, 500 µL of (3-aminopropyl)triethoxysilane (APTES, ABCR, Karlsruhe, Germany) diluted in 1 mL of anhydrous toluene were added to the suspension. The suspension was stirred in darkness at 400 rpm at 80 °C for 24 h. The nanoparticles were washed twice with 30 mL of pure ethanol and then dried at 30 °C under vacuum for 24 h, to obtain particles loaded with levofloxacin and functionalized with primary amine groups (MBGN-L-NH_2_). The number of APTES anchored to MBGN was determined to be around 5% (wt%) by thermogravimetric analysis. Based on this data, we proceeded to incorporate *N*-acetylcysteine (*N*-Acetyl-L-cysteine, Sigma-Aldrich, St. Louis, MO, USA) in 1:1 (APTES/NAC) molar ratio via carbodiimide chemistry (see Scheme 1) [37]. For this purpose, 0.263 g of NAC, 0.665 g of *N,N’*-dicyclohexylcarbodiimide (DCC, Aldrich, St. Louis, MO, USA) and 0.315 g of *N*-hydroxysuccinimide (NHS, Sigma-Aldrich, St. Louis, MO, USA) were mixed in 6 mL of *N,N*-dimethylformamide (DMF, Panreac, Barcelona, Spain) and stirred at 800 rpm at RT until a precipitate appeared. The filtrate was added to a suspension of 300 mg of nanoparticles that had been previously functionalized with APTES (MBGN-L-NH_2_) in 12 mL of DMF and stirred in darkness at 800 rpm at RT for 24 h. The resulting nanoparticles (MBGN-L-NAC) were then washed twice with 30 mL of pure ethanol and dried at 30 °C under vacuum for 24 h. Scheme 1 shows the synthetic strategy followed in this work, so that NAC is anchored through the carboxyl group, while the thiol (-SH) group remains unaltered on the nanoparticle surface. The same method was carried out with MBGN without levofloxacin, to obtain MBGN-NAC, a nanosystem with only NAC anchored to its surface by an amide bond with APTES.

### 2.4. Characterization Techniques

Transmission electron microscopy (TEM) was carried out using a JEOL 1400 microscope (JEOL Ltd., Tokyo, Japan), operating at 300 kV (Cs 0.6 mm, resolution 1.7 Å) equipped with an energy-dispersive X-ray spectrometer (EDS). Nitrogen adsorption/desorption isotherms were obtained using a Micromeritics 3Flex surface analyzer (Micromeritics, Norcross, GA, USA). Samples were previously degassed under vacuum for 72 h at 50 °C. The surface area was determined using the Brunauer–Emmett–Teller method (BET), the pore size distribution between 0.5 and 40 nm was determined from the adsorption branch of the isotherm by means of the Barret–Joyner–Halenda method (BJH), and the pore volume was determined from the amount of N_2_ adsorbed at a relative pressure of 0.95 [38]. Fourier transform infrared spectroscopy (FTIR) was carried out using a Nicolet Magma IR 550 spectrometer with a Golden Gate accessory (Thermo Electron Scientific Instruments LLC., Waltham, MA, USA) to use the attenuated total reflectance (ATR) sampling technique (64 scans, 4. resolution and 400–4000 cm^−1^ scan range). Thermogravimetric analysis (TGA) was carried out using a TG/DTA Seiko SSC/5200 thermobalance (Seiko Instruments, Chiba, Japan) between 50 °C and 600 °C at a heating rate of 5 °C/min, using aluminium crucibles and α-Al_2_O_3_ as reference. Elemental analysis was performed on an elemental microanalyzer LECO CHNS-932 3288 (LECO Instruments SL, Tres Cantos, Spain).

### 2.5. In Vitro Bioactivity Assay

In vitro bioactivity was evaluated as the capability of nanoparticles to form an apatite-like phase like the mineral component of bone when treated with a solution mimicking the ionic component of the human plasma. For this purpose, a suspension of 40 mg of MBGN, MBGN-L, and MBGN-L-NAC in 10 mL of filtered simulated body fluid (SBF) was incubated with orbital shaking at 100 rpm at 37 °C for 168 h. SBF pH 7.4 was prepared according to Kokubo [39] and then was filtered through 0.22 µm filters (Millipore, Burlington, MA, USA) to avoid bacterial contamination. The apatite nucleation and growth was analyzed by FTIR, TEM, and EDS analysis, as previously described, and the concentrations of Ca, P, and Si elements in the SBF were analyzed by inductively coupled plasma spectroscopy (ICP-OES) in an ICP-OES Agilent 5800 (Agilent Technologies, Santa Clara, CA, USA) with detection limits of 0.006 ppm (Ca), 0.012 ppm (P), and 0.007 ppm (Si).

### 2.6. Levofloxacin Release Study

A suspension of 10 mg/mL of MBGN-L and MBGN-L-NAC in a phosphate buffered saline solution (PBS) previously adjusted to pH 6.0 using HCl 1M, to mimic pH infection conditions, was incubated in darkness at 37 °C with orbital shaking at 100 rpm for 72 h. At each time point, the suspension was centrifuged, and fluorescence was measured using a FLUOstar Omega microplate reader version V6.20 Edition 4 (BMG Labtech, Ortenberg, Germany) exciting the sample at 355/20 nm and measuring fluorescence emission at 520 nm to determine the levofloxacin concentration. PBS pH 6.0 was then refreshed, and the 10 mg/mL suspension was incubated under the same conditions until the next measurement.

To determine the total amount of levofloxacin-loaded, a suspension of 2 mg/mL of MBGN-L and MBGN-L-NAC in HCl 1M was incubated in darkness at 37 °C and was stirred at 400 rpm for 72 h. The suspension was then centrifuged at 10,000 rpm for 15 min, and the supernatant fluorescence was measured using a FLUOstar Omega microplate reader (BMG Labtech, Ortenberg, Germany) exciting the sample at 355/20 nm and measuring fluorescence emission at 520 nm to determine the levofloxacin concentration.

In order to assess the effect of pH in the levofloxacin release, 10 mg/mL of MBGN-L and MBGN-L-NAC in PBS at pH 6.0 and at pH 7.4 were incubated in darkness at 37 °C with orbital shaking at 100 rpm for 30 h, measuring the levofloxacin released as explained above.

### 2.7. In Vitro Antimicrobial Evaluation

#### 2.7.1. Biofilm Formation and Treatment with Nanoparticles

The biofilm formation and treatment has been performed following previous works from our group [36]. Briefly, a suspension of *S. aureus* (ATCC 29213) was cultured in aseptic conditions in previously sterilized Todd–Hewitt broth (THB, Fluke, Buchs, Switzerland) with orbital shaking at 100 rpm at 37 °C and 5% CO_2_ until OD600 reached 0.4. The suspension was then diluted in sterilized THB supplemented with 1% sucrose and filtered through 0.22 µm filters (Millipore, Burlington, MA, USA) to reach a final concentration of 1 × 10^6^ CFU/mL, and 1 mL of this suspension was added to 24-well plates that were incubated for 48 h with orbital shaking at 100 rpm at 37 °C and 5% CO_2_ with medium refreshment every 24 h. Formed mature *S. aureus* biofilms were washed twice with PBS under aseptic conditions.

Since the amount of levofloxacin incorporated was different for MBGN-L and MBGN-L-NAC samples, the number of nanoparticles added to the biofilms were established so that the levofloxacin released after 24 h from both nanoparticles were 4.8 µg/mL. In this way, 1 mL of 200 µg/mL of MBGN, 200 µg/mL MBGN-L-NAC, 99.7 µg/mL of MBGN-L, and 4.8 µg/mL of levofloxacin (Levo) in THB 1% sucrose were added and incubated under previous conditions for 24 h. The treated biofilms were washed twice with PBS under aseptic conditions and then disaggregated with a pipette in 1 mL of PBS. Serial dilutions in PBS were made, and five drops of 10 µL of them were incubated in sterilized tryptic soy agar (TSA, Millipore, Burlington, MA, USA) for 24 h to determine the CFU/mL inside the biofilms treated.

#### 2.7.2. Live/Dead Bacteria Study

Nanoparticle-treated biofilms were washed twice with PBS and were incubated with 5 µM Syto9 and 30 µM propidium iodide (LiveDead *Bac*Light^TM^, Invitrogen, Waltham, MA, USA) in PBS for 15 min at RT in darkness, and then, calcofluor white stain (Fluka, Buchs, Switzerland) was added to reach a final concentration of 5 µg/mL. An Olympus FV1200 confocal laser microscope (Olympus, Tokyo, Japan) was used to analyze the stained biofilms exciting Syto9 at 480 nm and registering the emission at 500 nm, exciting propidium iodide at 490 nm and registering the emission at 600 nm, and exciting calcofluor at 405 nm and registering the emission range at 425–525 nm. ImageJ software version 1.54f (NIH, Bethesda, MD, USA) was used to determine the average of red pixels (dead bacteria, propidium iodide) green pixels (alive bacteria, Syto9), and blue pixels (mucopolysaccharide, calcofluor) in each 60× image taken.

### 2.8. In Vitro Cell Culture Studies with MC3T3-E1 Pre-Osteoblasts

#### 2.8.1. In Vitro Cell Proliferation Assay

3 × 10^4^ MC3T3-E1 murine pre-osteoblasts were seeded in 24-well plates with 1 mL of minimum essential medium alpha modification (alpha-MEM) containing 10% fetal bovine serum (FBS), 1% *L*-glutamine, and 1% penicillin/streptomycin (supplemented alpha-MEM; Gibco, Waltham, MA, USA). Cell cultures were treated with 1 mL of 200 µg/mL of MBGN, 200 µg/mL MBGN-L-NAC, and 99.7 µg/mL of MBGN-L in supplemented alpha-MEM for 3 and 24 h at 37 °C in a 5% CO_2_ atmosphere without medium renewing. The lower particle concentration for the MBGN-L sample was determined to maintain the same dose of levofloxacin released in 24 h with respect to MBGN-L-NAC, as explained above. These concentrations of nanoparticles and conditions were kept for the rest of cell culture assays. Cells were washed twice with PBS and were incubated with CCK-8 1% (Sigma-Aldrich, St. Louis, MO, USA) in supplemented alpha-MEM for 4 h at 37 °C in darkness in a 5% CO_2_ atmosphere. Absorbance was measured at 450 nm in a FLUOstar Omega microplate reader (BMG Labtech, Ortenberg, Germany).

#### 2.8.2. Determination of Reactive Oxygen Species (ROS)

5 × 10^4^ MC3T3-E1 murine pre-osteoblasts were seeded in 24-well plates with 1 mL of supplemented alpha-MEM. Cell cultures were treated with the nanoparticles for 3 and 24 h without medium renewing and then harvested using trypsin-EDTA 0.25% (Gibco, Waltham, MA, USA) for 3 min, which was neutralized with supplemented alpha-MEM. The suspension was centrifuged at 1200 rpm for 5 min, and the resulting pellet was resuspended in 7 µM DCFH-DA (Sigma-Aldrich, St. Louis, MO, USA) in PBS and incubated for 30 min at 37 °C in a 5% CO_2_ atmosphere in darkness. Cells were analyzed using a FACScalibur flow cytometer (Becton Dickinson, Franklin Lakes, NJ, USA) exciting DCFH at 488 nm and registering the emission at 530/30 nm. Untreated cells without DCFH-DA were used to discard autofluorescence and to select gating, and untreated cells with DCFH-DA were used as the control of intracellular ROS.

#### 2.8.3. In Vitro Cell Differentiation Assay

3 × 10^4^ MC3T3-E1 murine pre-osteoblasts were seeded in 24-well plates with 1 mL of supplemented alpha-MEM. Cell cultures were treated with nanoparticles for 24 h, were washed twice with PBS, and were subsequently cultured for 8 and 14 days, refreshing medium without nanoparticles every 3 days. Alkaline phosphatase (ALP) activity was measured using an ALP activity kit (Spinreact, St. Esteve de Bas, Spain) and was related to the total protein produced by the cells using a Total Protein kit (Spinreact, St. Esteve de Bas, Spain), according to the manufacturer’s protocol.

#### 2.8.4. In Vitro Mineralization Assay

1 × 10^5^ MC3T3-E1 pre-osteoblasts were seeded in six-well plates with 2 mL of supplemented alpha-MEM. Cell cultures were treated with nanoparticles for 24 h, were washed twice with PBS, and then cultured for 21 days, refreshing medium without nanoparticles every 3 days. After two washes with PBS, cells were fixed with glutaraldehyde 10% (Sigma-Aldrich, St. Louis, MO, USA) for 1 h at 4 °C and then, Alizarin Red (Sigma-Aldrich, St. Louis, MO, USA) 40 mM pH 4.2 solution was added for 45 min at RT in darkness to stain the calcium deposits. After extensive washing with tap water, the deposits were solubilized with 50% acetic acid and quantified by measuring the absorbance at 570 nm in a FLUOstar Omega microplate reader (BMG Labtech, Ortenberg, Germany).

#### 2.8.5. Confocal Laser Scanning Microscopy

3 × 10^4^ MC3T3-E1 murine pre-osteoblasts were seeded in coverglass coverslips contained in 24-well plates with 1 mL of supplemented alpha-MEM. Cell cultures were treated with the nanoparticles suspended in supplemented alpha-MEM for 24 h at 37 °C in a 5% CO_2_ atmosphere, then were washed twice with PBS and were cultured for 14 days at 37 °C in a 5% CO_2_ atmosphere, refreshing medium without nanoparticles every 3 days. Cells were fixed using paraformaldehyde 4% in PBS for 24 h at 4 °C and were permeabilized with 1 mL of Triton X-100 0.1% (Sigma-Aldrich, St. Louis, MO, USA) and Bovine Serum Albumin 1% (BSA, Sigma-Aldrich, St. Louis, MO, USA) in PBS for 1 h at 4 °C. Finally, nuclei were stained using DAPI 3 µM (Sigma-Aldrich, St. Louis, MO, USA), cytoskeleton was stained using Phalloidin Atto 565 0.14 nM (Sigma-Aldrich, St. Louis, MO, USA) and coverslips were mounted using ProLong Gold anti-fade reagent (Invitrogen, Waltham, MA, USA). Cells were visualized in an Olympus FV1200 confocal laser microscope (Olympus, Tokyo, Japan) exciting DAPI at 405 nm registering the emission at 420–480 nm, and exciting Atto 565 at 564 nm registering the emission at 590 nm.

### 2.9. Statistical Analysis

Data are expressed as media ± standard deviation of at least triplicates for every condition. Statistical analysis was performed using Statistical Package for the Social Sciences (2013 IBM SPSS STATISTIC) version 28, performing ANOVA and Levene’s tests with either Scheffé or Games–Howell post hoc evaluation to assess the difference between the groups. *p* < 0.05 was considered as statistically significant.

## 3. Results

### 3.1. Synthesis and Characterization of the Nanosystems

**Figure 1:**
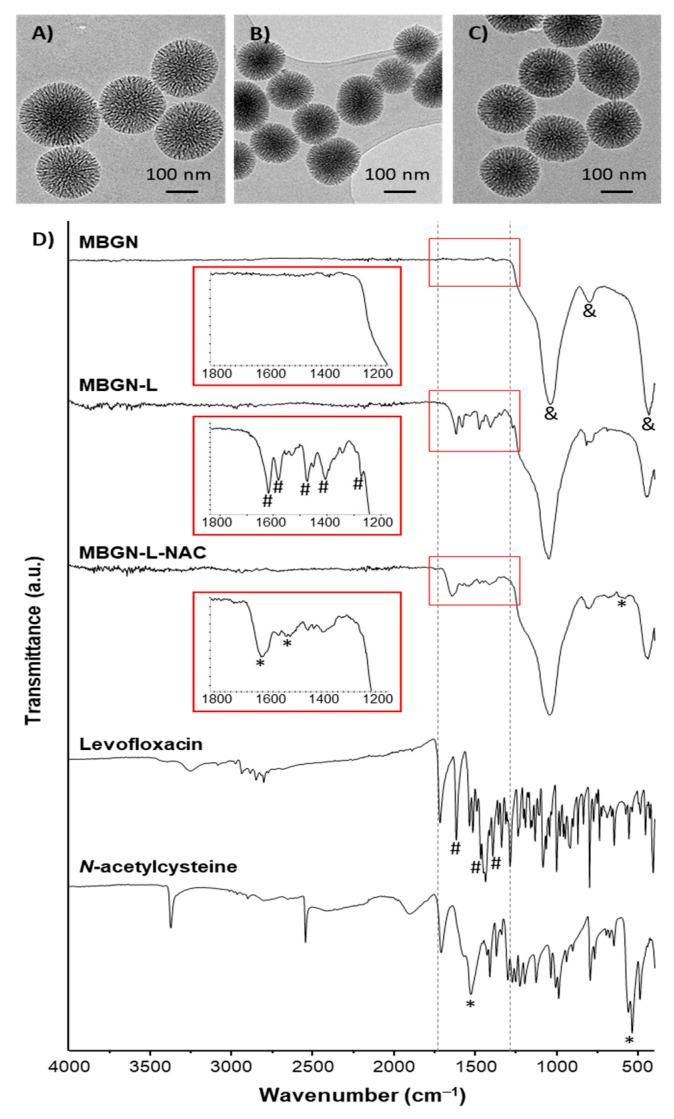
Physicochemical characterization of the different materials synthesized. TEM images of (**A**) MBGN, (**B**) MBGN-L, and (**C**) MBGN-L-NAC samples. (**D**) FTIR spectra of the obtained materials. “&” symbol indicates the bands assigned to silica mesoporous framework; “#” symbol indicates the levofloxacin bands, and “*” symbol corresponds to the *N*-acetylcysteine anchoring via amide bond.

The morphology and porous structure of the synthesized nanoparticles (MBGN) were determined by TEM (Figure 1A). MBGN shows a spherical morphology with a diameter around 150 nm and a radial porous structure. The EDS spectra acquired during the TEM observation showed a composition of 82.5 SiO_2_-17.5 CaO (% mol). After loading with levofloxacin, MBGN-L, (Figure 1B) and functionalization with NAC, MBGN-L-NAC, (Figure 1C) nanoparticles did not undergo morphological modifications or collapse of the porous structure, maintaining similar characteristics compared to MBGN.

The adsorption of levofloxacin, as well as the anchoring of NAC, were confirmed by FTIR (Figure 1D). The spectrum of MBGN exhibits absorbance bands at 1080 cm^−1^, 798 cm^−1^, and 430 cm^−1^ corresponding to typical absorption bands of the Si-O-Si bond of silica mesoporous framework. The weak absorption band approximately 1080 cm^−1^ can be assigned to the Si-O-Si asymmetric stretching vibrations of SiO_4_ tetrahedral. The band at 798 cm^−1^ indicates the bending vibrations of –OH on Si-OH groups typical of mesoporous glass, while the band at 430 cm^−1^ represented Si-O bending vibrations of the silica network [40]. The presence of Si-OH is critical for the subsequent functionalization of MBGN, as Si-OH groups are the chemical groups used for the incorporation of amino groups by silanization.

After incorporation of levofloxacin (MBGN-L) absorbance bands appear at 1617 cm^−1^, 1582 cm^−1^, 1476 cm^−1^, and 1407 cm^−1^, corresponding to the stretching of levofloxacin alkane bonds, and another absorbance band appears at 1270 cm^−1^ corresponding to C-N stretching in aromatic amines [41]. The appearance of absorption bands assigned to levofloxacin evidences the presence of this antibiotic within the porous structure of the nanoparticles. Then, APTES was grafted on the nanoparticles using a post-synthesis method [42] followed by NAC anchoring via carbodiimide-mediated, as described in Scheme 1 [37]. Finally, after NAC anchoring (MBGN-L-NAC), the absorbance bands of the levofloxacin described above make it difficult to distinguish the *N*-acetylcysteine in the spectrum by overlapping. Although it is observed a broad band at 1640 cm^−1^ and another band at 1530 cm^−1^ corresponding to the amide bond between the primary amines on the nanoparticles surface and the carboxylic acid group of NAC molecules, confirming the covalent anchorage of the *N*-acetylcysteine. The band observed at 659 cm^−1^ is assigned to SH bending of NAC [43]. These absorption bands indicate that NAC has covalently anchored to the amine-functionalized surface through the carboxylic group, leaving the thiol group available to exert disaggregation activity on the bacterial biofilm.

The incorporation of levofloxacin and the anchoring of NAC were also assessed by elemental chemical analysis (Table 1). MBGNs show a very low residual percentage of C, indicating the calcination at 600 °C of the surfactant. Nanoparticles loaded with levofloxacin (MBGN-L) show an increase of C and N atoms with respect to MBGN, which also occurs for the MBGN-L-NAC sample. The presence of S in MBGN-L-NAC up to approximately 1% confirms the presence of thiol groups of NAC in the material in agreement with FTIR results.

Thermogravimetric analysis was used to quantify the percentage of levofloxacin in MBGN-L and MBGN-L-NAC (Figure S1 in the Supplementary Material). MBGN-L shows a loss of 21.5% by mass corresponding to the amount of levofloxacin, while MBGN-L-NAC shows a loss of 19% of organic matter, which would correspond to the thermo-oxidative decomposition of levofloxacin and NAC. The higher amount of organic matter present in MBGN-L compared to MBGN-L-NAC confirmed that a significant fraction of levofloxacin was leached out during the NAC functionalization process to obtain MBGN-L-NAC. Therefore, we decided to carry out a levofloxacin release study under acidic conditions using HCl 1M as solvent, forcing the total release of the drug during 3 days, and analyzing the amount of levofloxacin by fluorescence. The total levofloxacin content was 23.12% (*w*/*w*) in MBGN-L and 5.86% (*w*/*w*) in MBGN-L-NAC.

The textural properties were studied by N_2_ adsorption/desorption analysis (Figure S2 in Supplementary Material). MBGN, MBGN-L, and MBGN-L-NAC exhibit type IV isotherms, with a significant increase of nitrogen adsorbed at intermediate relative pressures. Table 2 shows surface area, pore size, and pore volume values. MBGN has a high surface area and a pore size and volume in the range of values of mesoporous nanomaterials. After levofloxacin adsorption (MBGN-L), pore volume and surface area are significantly reduced to almost a 50% respect to the MBGN values, signaling the efficient confinement of the levofloxacin molecules into the mesoporous cavities. The incorporation of NAC (MBGN-L-NAC) leads to a reduction in pore size, volume, and surface area. The progressive decrease of textural values, such as surface area and pore volume, evidence that the filling of the pores with levofloxacin and the anchoring of NAC on the accessible surface of MBGNs has been successfully performed by the synthesis scheme proposed in Scheme 1, in agreement with the results obtained by FTIR spectroscopy.

### 3.2. In Vitro Bioactivity Assay

**Figure 2:**
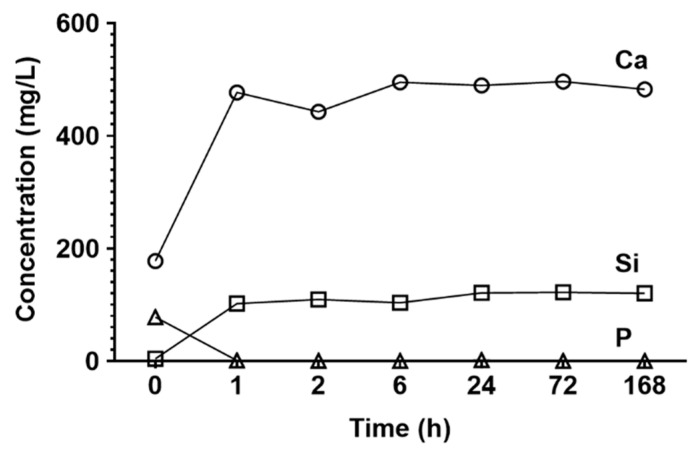
Atom concentrations of Ca, Si, and P in SBF from a suspension of MBGN during 168 h.

The study of compositional changes in the SBF (Figure 2) confirms the release of Ca^2+^ ions to the medium during the first hour of soaking of the nanoparticles, reaching concentrations of up to 500 ppm whereas the concentration of phosphorus decreases simultaneously until the almost total depletion of this element. On the other hand, the release of silica indicates a slight degradation of the nanoparticles. Initial pH of SBF was 7.4 and rose up to 8.1 after 7 days of soaking.

**Figure 3:**
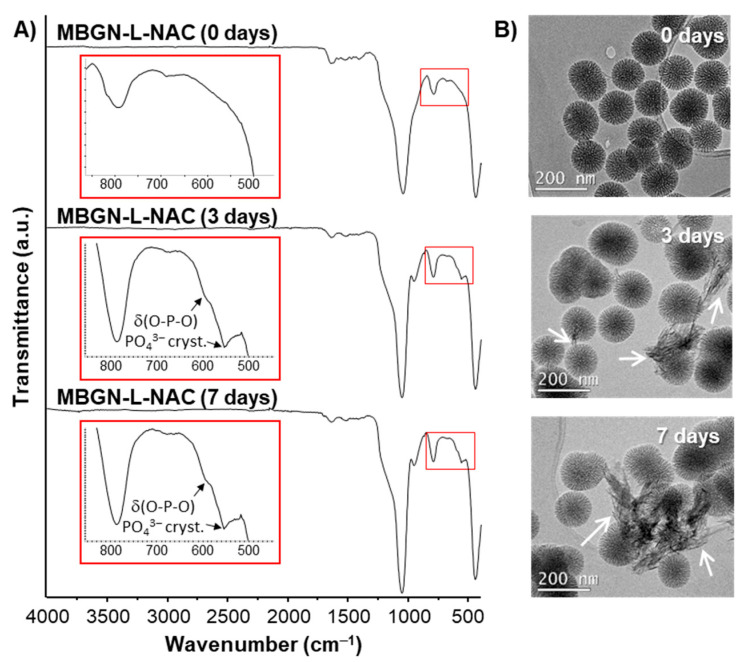
In vitro bioactivity testing of MBGN-L-NAC after being soaked in SBF for 0, 3, and 7 days. (**A**) FTIR spectra and (**B**) TEM images corresponding to MBGN-L-NAC after soaking in SBF for different times. The white arrows show the needle-like calcium phosphate crystals formed.

The in vitro bioactivity was also studied through the changes produced on the surface of the nanoparticles in contact with SBF. After 3 and 7 days in SBF, the FTIR spectra for the MBGN-L-NAC sample show the presence of a doublet located at 580 and 610 cm^−1^ corresponding to the bending mode of the O-P-O bonds of the PO_4_^3−^ ion in a crystalline environment (Figure 3A). These bands indicate the formation of needle-like calcium phosphate crystals on the MBGN-L-NAC surface, as shown by TEM images after the same periods of SBF treatment (Figure 3B). EDS analysis indicated an increase in Ca and P elements of 10.32% and 7.09%, respectively. In addition, MBGN and MBGN-L samples showed a very similar behavior, although the formation of calcium phosphate crystals occurred to a greater extent on the MBGN sample (Figures S3 and S4 in Supplementary Material). Comparing the FTIR spectra of MBGN-L-NAC after SBF soaking for 7 days with MBGN-NAC (nanoparticles with only NAC anchored, Figure S5 in the Supplementary Material), we observed that both nanosystems exhibit absorption bands at 1640 and 1530 cm^−1^ corresponding to amide bond, thus confirming the permanence of NAC in both nanosystems during the bioactive process.

### 3.3. Levofloxacin Release Study

**Figure 4:**
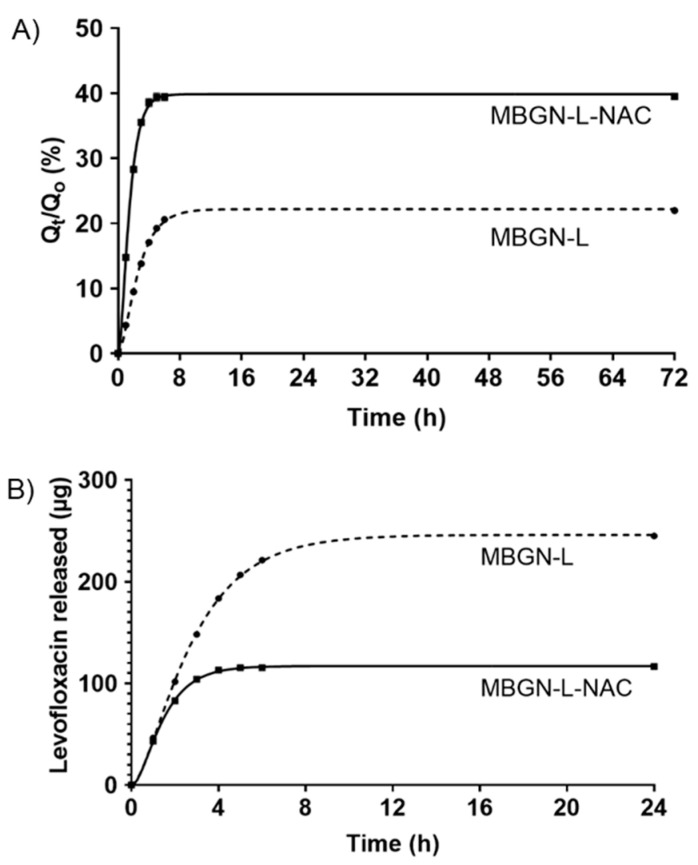
“In vial” cumulative levofloxacin release profiles from MBGN-L and MBGN-L-NAC nanosystems at pH 6.0 in PBS during 72 h (**A**). Q_T_ refers to the amount of released levofloxacin versus time, and Q_0_ refers to the total amount of loaded levofloxacin into the material. (**B**) Amount of levofloxacin released from both materials during the first 24 h.

Figure 4A shows the in vial release profiles of levofloxacin from MBGN-L and MBGN-L-NAC as a function of time. Both exhibit a typical profile of drug release from mesoporous silica nanoparticles, characterized by a high initial release (burst effect) followed by a more sustained release over time [44]. Both release profiles can be adjusted to first-order kinetic model by introducing an empirical non-ideality factor (*d*) to give the following equation [44]:

(1) $$Q_{t}/Q_{0}=A{\left(1-e^{\left(-kt\right)}\right)}^{d}$$

being *Q_t_/Q*_0_ the percentage of levofloxacin release at *t*, *A* the maximum amount of drug released, and *k* the release kinetic constant. The value *d* is comprised between 1 for materials that obey first-order kinetics, and 0, for the materials that release the loaded drug in the very initial time of the assay. The parameters of the kinetics fitting (Table 3) display that the slowest levofloxacin release occurs from MBGN-L matrix (*k* = 0.16 h^−1^), showing a *d* = 1, which indicates a first-order release kinetics and release approximately 30% after 72 h. For the case of MBGN-L-NAC there is a very slight variation from the ideal first-order kinetics, with a *d* value of 0.9. In this case, release rate of MBGN-L-NAC is faster than that of MBGN-L, with *k* values of 0.52 h^−1^ and a total release of approximately 43% after 72 h.

Taking into account these results, we have designed the antimicrobial activity studies until 24 h, ensuring the maximum amount of levofloxacin released for each sample. Moreover, we have calculated the dosage of levofloxacin released from 5 mg of each sample in 0.5 mL of PBS pH 6.0 during 24 h (Figure 4B), evidencing a total amount of released levofloxacin of 238.4 µg and 116.7 µg for MBGN-L and MBGN-L-NAC, respectively. These results show that the amount of levofloxacin released by MBGN-L is twice the amount released by MBGN-L-NAC after 24 h. In order to keep the doses of levofloxacin constant, the amounts of MBGN-L and MBGN-L-NAC for the subsequent microbiological studies were added considering this difference. Previous reports indicate that the minimum bactericidal concentration (MBC) of levofloxacin against *S. aureus* is 4 µg/mL [45]. Therefore, the different nanoparticles concentrations were adjusted to release a slightly higher drug concentration, i.e., 4.8 µg/mL.

For comparison purposes, levofloxacin release was also studied at pH 7.4 (Figure S6 in the Supplementary Material). At this physiological pH, the drug release rate is lower compared to the drug release at pH 6.0, pointing out that the acidic media commonly produced in infected tissue favor levofloxacin release.

### 3.4. In Vitro Antimicrobial Evaluation

Mature biofilms of *S. aureus* were treated for 24 h with different nanoparticles, adjusting the number of materials to provide the same concentration of released levofloxacin (4.8 µg/mL) after this period. This standardization was carried out to observe the effect of the NAC present in the samples on the destruction of the biofilm, eliminating the possible effect produced by a greater release of antibiotic [46].

**Figure 5:**
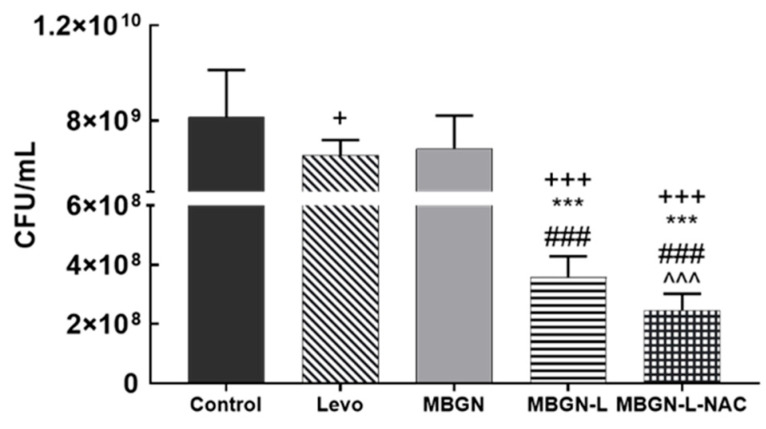
Viability of mature bacterial biofilm after 24 h of treatment with the different synthesized nanosystems. Statistical significance: + *p* < 0.05 vs. Control, +++ *p* < 0.005 vs. Control, *** *p* < 0.005 vs. Levo, ### *p* < 0.005 vs. MBGN, ^^^ *p* < 0.005 vs. MBGN-L.

The biofilms after treatment were disaggregated and diluted to recover the bacteria that survived the different treatments. The CFU/mL (colony-forming units) remaining in these biofilms are shown in Figure 5. MBGN-L and MBGN-L-NAC significantly reduce the viability of the biofilm compared to Levo and MBGN conditions. When comparing MBGN-L with respect to MBGN-L-NAC, we observe that the MBGN-L-NAC condition significantly reduces the biofilm viability.

**Figure 6:**
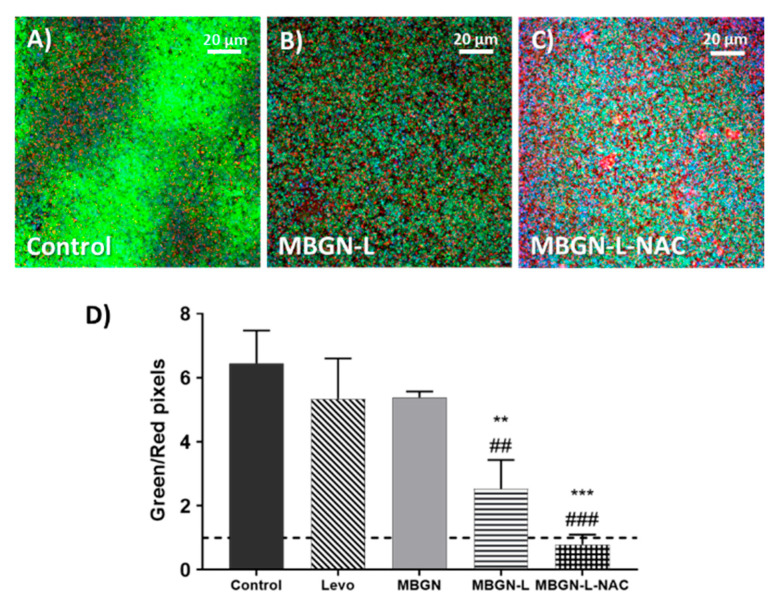
Confocal studies after treating the biofilms for 24 h with the different nanosystems. Confocal images of the (**A**) Control, (**B**) MBGN-L, and (**C**) MBGN-L-NAC-treated biofilms. (**D**) Ratio of Alive vs. Dead bacteria. (** *p* < 0.01 vs. Levo, *** *p* < 0.005 vs. Levo, ## *p* < 0.05 vs. MBGN, ### *p* < 0.005 vs. MBGN).

Treated biofilms were stained with the *Bac*Light^TM^ Live/Dead counting kit (Figure 6A). The ratio of green and red pixels, observed by confocal microscopy, (live and dead bacteria, respectively) is shown in Figure 6B. The MBGN-L and MBGN-L-NAC conditions significantly reduce the live/dead ratio compared to the Levo and MBGN conditions, and the MBGN-L-NAC treatment is the only one that reduces the ratio below 1 (shown in Figure 6B).

### 3.5. In Vitro Cell Culture Studies

**Figure 7:**
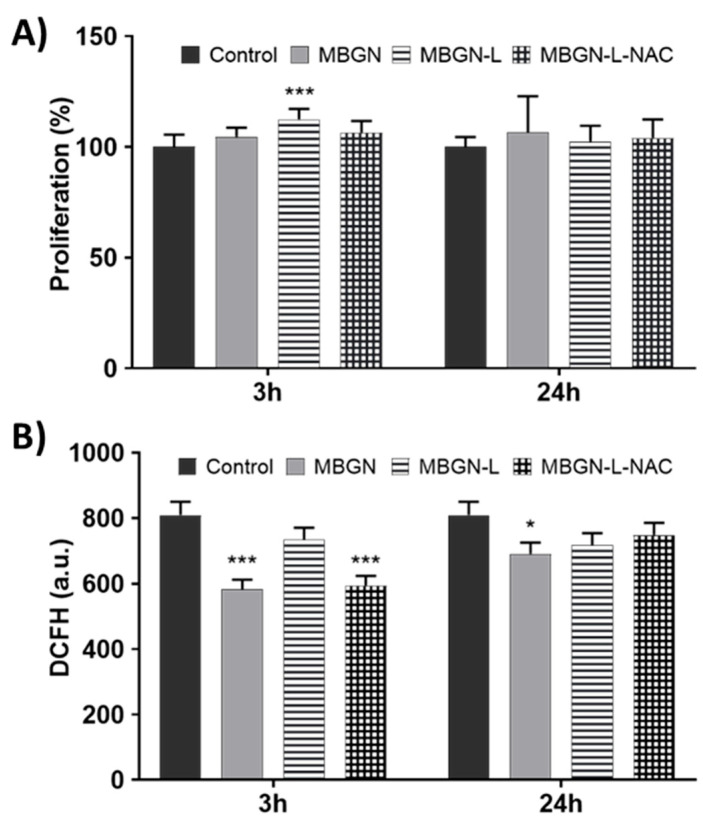
In vitro biocompatibility of different obtained materials in MC3T3-E1 murine pre osteoblasts after different time periods (3 and 24 h). (**A**) Cell proliferation measured with CCK-8 and (**B**) Intracellular ROS content analysed by flow cytometry. Statistical significance: * *p* < 0.05 vs. Control, *** *p* < 0.005 vs. Control.

The biocompatibility of the different nanoparticles was tested in MC3T3-E1 murine pre-osteoblast culture in vitro. Regarding the number of nanoparticles added to the cell culture, the same conditions with respect to the in vitro antimicrobial evaluation were used. Figure 7A shows that the proliferation rate referred to in the control is maintained in all the conditions tested, even enhanced in the 3-h contact with the MBGN-L condition. Figure 7B shows that all the nanosystems reduce the ROS intracellular content compared to the control at a 3-h contact, with statistically significant differences for MBGN and MBGN-L-NAC. This reduction of intracellular ROS content is also observed after 24 h, although only the MBGN condition significantly reduces this parameter compared to the control.

**Figure 8:**
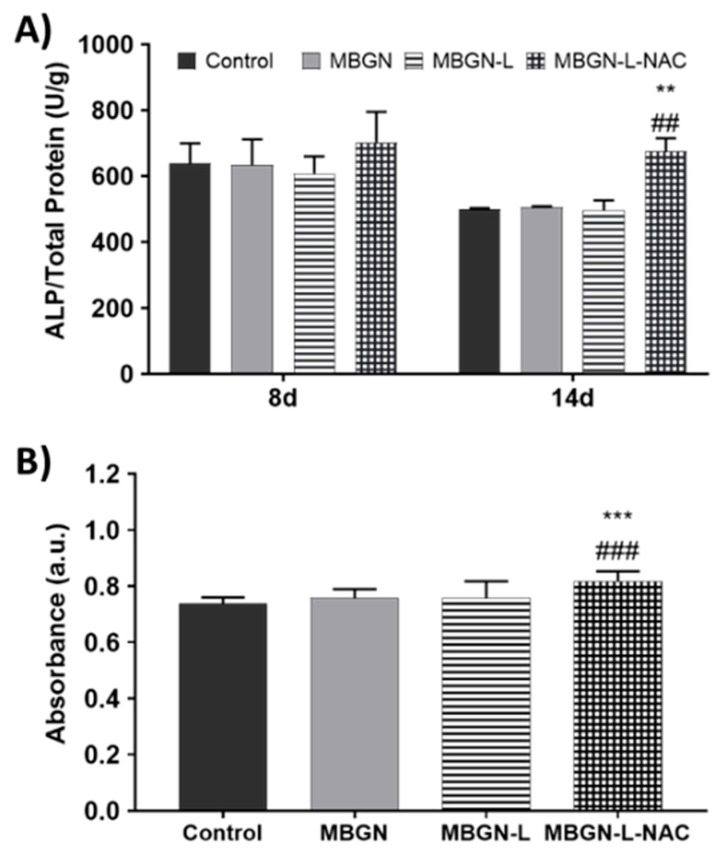
In vitro cell-differentiation studies on MC3T3-E1 murine pre-osteoblasts in contact with the different synthesized nanomaterials. (**A**) ALP activity measured at different times (8 and 14 days of culture), after 24 h of contact with the nanomaterials and (**B**) Mineralization of calcium deposits at 21 days of culture, after 24 h of contact with the nanomaterials. Statistical significance: ** *p* < 0.01 vs. Control, *** *p* < 0.005 vs. Control, ## *p* < 0.01 vs. MBGN, ### *p* < 0.005 vs. MBGN.

Figure 8A shows the alkaline phosphatase activity measurements. No significant differences can be observed at 8 days of cell culture, while at 14 days of cell culture, the pre-osteoblasts treated for 24 h with MBGN-L-NAC condition show a significant increase in alkaline phosphatase activity compared to control and MBGN conditions. Figure 8B shows the mineralization of calcium deposits after 21 days of differentiation. Treatment of pre-osteoblasts for 24 h with MBGN-L-NAC leads to a slightly but statistically significant increase of calcium mineralization compared to control and MBGN conditions.

**Figure 9:**
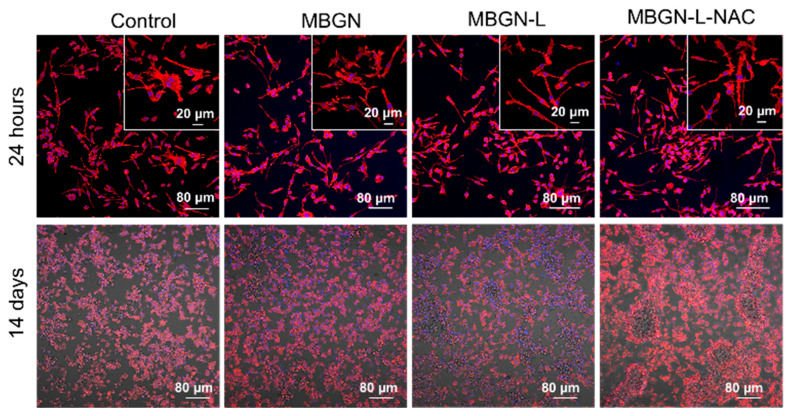
Confocal and phase contrast microscopy images of MC3T3-E1 cells treated with MBGN, MBGN-L, and MBGN-L-NAC for 24 h and after 14 days of culture post-treatment. Staining: Nuclei (DAPI, blue), Cytoskeleton (Phalloidin, red). The selected confocal plane is the closest to the coverglass.

Figure 9 shows the behavior of the MC3T3-E1 culture after being in contact with the different nanosystems for 24 h and 14 days. After 24 h, we observed similar cell proliferation in all conditions tested, according to the results presented in Figure 7A. Likewise, the cytoskeletons for the pre-osteoblasts treated with the different particles are like the control, showing a pyramidal shape typical of pre-osteoblasts with numerous filopodia due to the proliferation phase in which they are found. At 14 days, pre-osteoblasts start to form some clusters in the Control conditions (Movie S1 in Supplementary Material), and cells acquire a cuboidal shape. Cells treated with MBGN and MBGN-L reproduce this behavior. However, pre-osteoblasts in contact with MBGN-L-NAC form significantly taller and wider dome-shaped cell clusters, reaching dimensions of up to 60 μm in height (Movie S2 in Supplementary Material).

## 4. Discussion

*S. aureus* is one of the main pathogens causing osteomyelitis associated with periprosthetic joint infections (PJI). Like many other pathogens, *S. aureus* forms a biofilm in the area where the implant is anchored to the bone, eroding the local tissue, leading to a decrease in implant support and, if left untreated, can lead to septicemia [1,47,48,49]. PJIs are commonly treated with tissue debridement combined with prolonged antibiotic treatment and, depending on the condition of the implant, it is either retained or replaced. Single-antibiotic treatment is effective only in the early stages of infection.

In this work, we have designed radial mesoporous bioactive glass nanoparticles (MBGN) with antibacterial and osteogenic properties. The high surface area and porosity of the MBGN facilitates the incorporation of levofloxacin into the mesoporous system, so that loading of up to 23 wt.% is achieved. According to the literature [50,51], levofloxacin molecules would bind to the MBGNs via hydrogen bonds with the silanol (Si-OH) groups on the surface of our silica-based nanoparticles. This strong interaction likely explains the incomplete release of the antibiotic, as only 21% of the total amount of levofloxacin was released after 72 h in aqueous medium at pH 6.0, and also explains the faster release rate of levofloxacin when comparing pH 6.0 to pH 7.4. As previously stated [52], at pH 7.4 levofloxacin molecule has a zwitterionic form that favors the formation of hydrogen bonds with the silanol groups of the nanoparticles, but at a more acidic pH, levofloxacin molecule acquires a cationic form, decreasing the hydrogen bond interaction and leading to a higher levofloxacin release. This result indicates that only a small fraction of the adsorbed drug is released early, while most of the hydrogen-bonded drug molecules remain retained and would only be available after further degradation of the nanoparticles.

The incorporation of NAC in MBGN-L-NAC was designed to expose the thiol functional group (-SH) to the surrounding medium to maintain mucolytic activity against bacterial biofilm. This requires prior functionalization with APTES, as shown in Scheme 1. APTES provides amine groups and acts as a linker to covalently attach the NAC molecules to the nanoparticles via amide bonds through carbodiimide chemistry [37]. This surface modification leads to significant changes in the nanoparticles as a drug-delivery system. Levofloxacin is firmly bound to MBGN via hydrogen bond interaction with silanol groups on the surface. Following functionalization with APTES and subsequent addition of NAC, there is a decrease in the density of silanol groups, resulting in weaker interactions in MBGN-L-NAC compared to MBGN-L. Consequently, levofloxacin releases more rapidly from MBGN-L-NAC, despite the lower amount of drug contained within.

Antibacterial assays show that incorporation of levofloxacin into MBGN is a highly effective strategy to treat *S. aureus* infection even when mature biofilm has developed. Our results show that treatment of the mature biofilm with free-levofloxacin is ineffective, as it leads to a minimal decrease in bacterial viability compared to the control group. This is explained by the effectiveness of the antibiotic resistance provided by the already-formed biofilm.

The incorporation of levofloxacin into MBGN-L significantly decreases the viability of the *S. aureus* biofilms. This suggests that MBGNs have a disruptive effect on the EPS of the biofilm. Although this disruptive effect would not affect the viability of the pathogen, it does make it easier for levofloxacin to reach it more effectively. In the case of MBGN-L-NAC, the disruptive capacity against the biofilm is significantly increased with respect to MBGN-L, mainly due to the mucolytic activity of the thiol (-SH) groups of NAC [53,54,55]. Consequently, the bactericidal capacity of the nanosystem increases, reaching a live/dead ratio of less than 1 for MBGN-L-NAC-treated biofilms. Our results indicate that MBGNs are excellent vehicles for transporting levofloxacin, avoiding the bacterial resistance provided by the biofilm. When the nanoparticles are functionalized with NAC, the bactericidal efficacy increases significantly, due to the deterioration of the biofilm.

Integral treatment of bone infection involves regeneration of damaged bone tissue in addition to elimination of the pathogen. The usage of a single platform capable of performing this combined therapy would be very advantageous for different clinical scenarios such as periodontal defects, bone regeneration in infected open fractures, or bone prosthesis infection. In the latter case, bone regeneration would restore the peri-implant tissue, thus avoiding loosening and replacement of the prosthesis. In this sense, the incorporation of NAC further enhances its bone-regenerative properties. As evident from in vitro biomineralization assays with SBF, MBGN-L-NAC nucleates and grows nanocrystalline hydroxycarbonate apatite very similar to the mineral component of bone and teeth. Our studies evidence that the Ca^2+^ ion concentration reached after several hours of soaking the nanoparticles in the SBF is a consequence of the release of Ca^2+^ ions into the medium and the depletion of this element in the SBF by the crystallization of the calcium phosphate phase. This biomimetic process is highly dependent on the textural properties of MBG [56,57]. The incorporation of NAC does not inhibit the in vitro bioactivity of the nanoparticles, nor does it affect cell viability, as clearly observed in the MC3T3-E1 proliferation results, which are very similar to those of the control.

NAC also promotes the osteoinductive behavior of MBGN-L-NAC. Our results demonstrate an enhanced expression of ALP and mineralization in osteoprogenitor cells treated with MBGN-L-NAC, pointing out that despite the covalent bonding to MBGN, this compound is able to exert its osteogenic properties. Previous studies suggest that NAC can lead to enhanced differentiation of osteoprogenitor cell via up regulation of Wnt 5a [58], as well as upregulation of bone-related gene markers such as collagen I, osteopontin, and osteocalcin [59].

It is well-known that osteoblasts develop through well-differentiated stages: proliferation, synthesis and maturation of the matrix, and mineralization. Between the first and second stages, the osteoblasts will decrease their proliferative activity and organize themselves into mineralization nodules: multilayered clusters of cells that will synthesize ALP and will secrete a collagenous matrix [60]. As shown in Figure 9 and Movie S2 in the Supplementary Material, pre-osteoblast culture in contact with MBGN-L-NAC creates multilayered cell nodules, which leads to an increase in the parameters of differentiation to mature osteoblast, thus confirming the osteogenic properties of NAC even after being covalently anchored to MBGNs.

Finally, it must be highlighted that ROS content is closely related to the stress state of the cell, leading to apoptosis or carcinogenesis [61], and has direct implications in immunology [33]. In this regard, in situations of infection, low ROS content is required for regeneration of damaged tissue [34]. MBGN with and without NAC seem to present an interesting feature as ROS scavengers in the early stages of cell culture, since a decrease in intracellular ROS is observed after 3 h in contact with osteoprogenitor cells. In the case of MBGN-L-NAC, the ROS scavenging properties can be explained by the antioxidant properties of NAC [62], together with the ability of MBG nanoparticles to decrease ROS content [63]. It is becoming increasingly recognised that ROS also mediate important physiological functions. Regardless of the sources of ROS, it has been shown that osteogenic differentiation is partly ROS dependent [64]. ROS are short-lived oxygen-containing molecules that display high chemical reactivity towards DNA, RNA, proteins, and lipids. Mitochondrial complexes I and III, and the NADPH oxidase isoform NOX4, are major sources of ROS production during mesenchymal stem cells (MSC) differentiation. ROS are thought to interact with several pathways that affect the transcription machinery required for MSC differentiation, including the Wnt, Hedgehog, and FOXO signaling cascades [65,66]. On the other hand, elevated levels of ROS, defined as oxidative stress, lead to arrest of the MSC cell cycle and apoptosis. Tightly regulated levels of ROS are therefore critical for MSC terminal differentiation [67]. In in vivo assays, the targeted effects of oxidative distress in mitochondria of cells of the osteoblast lineage have been examined with models of SOD2 depletion. In these assays, bone from SOD2-deficient mice exhibits a higher number of senescent cells [68], which supports the idea that elevated oxidant levels cause cellular senescence. These findings suggest that an increase in mitochondrial ROS in cells of the osteoblast lineage negatively impacts bone formation and, indirectly, stimulates bone resorption.

## 5. Conclusions

In this work, a novel nanosystem composed of mesoporous bioactive glass nanoparticles loaded with levofloxacin and functionalized with *N*-acetylcysteine has been achieved. The nanosystem combines the bone-regenerative properties of MBGNs, the antimicrobial effect of levofloxacin, and the mucolytic, osteogenic, and ROS-scavenging ability of *N*-acetylcysteine.

This nanoplatform is able to significantly reduce the viability of mature *S. aureus* biofilms. Furthermore, this nanosystem is biocompatible with MC3T3-E1 murine pre-osteoblasts and promotes their differentiation towards an osteoblast-mature phenotype. This nanosystem can be proposed as an alternative for the treatment of periprosthetic joint infections caused by *S. aureus.*

## Figures and Tables

**Table 1:** Elemental chemical analysis of the different nanosystems synthesized.

	%C	%N	%S
MBGN	0.7 ± 0.4	<0.5	-
MBGN-L	11.0 ± 0.4	2.2 ± 0.3	-
MBGN-L-NAC	10.5 ± 0.4	2.9 ± 0.3	1.1 ± 0.4

**Table 2:** Textural parameters derived from nitrogen-adsorption measurements of the different synthesized nanoparticles.

	Surface Area [m^2^/g]	Pore Size [nm]	Pore Volume [cm^3^/g]
MBGN	348.9	8.0	0.6
MBGN-L	206.9	8.5	0.3
MBGN-L-NAC	144.8	6.7	0.2

**Table 3:** Parameters and *R*^2^ of the adjustment to the Chapman equation of the release of levofloxacin by MBGN-L and MBGN-L-NAC during the first 6 h.

	*A*	*k*	*d*	*R* ^2^
MBGN-L	31.76	0.16	0.9	0.995
MBGN-L-NAC	42.75	0.52	1	0.990

## Data Availability

The raw data supporting the conclusions of this article will be made available by the authors on request.

## Supplementary Materials

The following supporting information can be downloaded at: https://www.mdpi.com/article/10.3390/pharmaceutics17040519/s1, Figure S1: Thermogravimetric analysis of the different synthesized nanoparticles; Figure S2: Nitrogen adsorption-desorption isotherm linear plots of the synthesized nanoparticles. (**A**) MBGN; (**B**) MBGN-L and C) MBGN-L-NAC isotherm linear plots; Figure S3: Bioactivity of MBGN in contact with SBF. (**A**) FTIR of the MBGN in contact with SBF for 0, 1 h, 2 h, 6 h, 24 h, 3 days, and 7 days. (**B**) TEM micrographs of MBGN in contact with SBF for 0, 3, and 7 days. (**C**) EDS spectra of MBGN in contact with SBF for 0, 3, and 7 days; Figure S4: Bioactivity of MBGN-L in contact with SBF. (**A**) FTIR of the MBGN-L in contact with SBF for 0, 3, and 7 days. (**B**) TEM micrographs of MBGN-L in contact with SBF for 0, 3, and 7 days. (**C**) EDS spectra of MBGN-L in contact with SBF for 0, 3, and 7 days; Figure S5: FTIR spectra of MBGN-NAC (up) and MBGN-L-NAC soaked in SBF for 7 days (down); Figure S6: Comparative levofloxacin release between pH 6.0 and pH 7.4 in (**A**) MBGN-L and (**B**) MBGN-L-NAC; Movie S1; Movie S2.
