# Supplementary material for: Osteogenic and Antibacterial Response of Levofloxacin-Loaded Mesoporous Nanoparticles Functionalized with N-Acetylcysteine
Source: Pharmaceutics. 2025 Apr 15;17(4):519. doi: 10.3390/pharmaceutics17040519 (PMC12030205; doi:10.3390/pharmaceutics17040519)
Supplement: Supplementary file 1 [file pharmaceutics-17-00519-s001.zip › pharmaceutics-3531213-supplementary-final/pharmaceutics-3531213-supplementary-final.docx]

**Supporting information**

**Osteogenic and antibacterial response of levofloxacin loaded mesoporous nanoparticles functionalized with N-acetylcysteine** Alberto Polo-Montalvo^1,3^, Natividad Gómez-Cerezo^1,4^, Mónica Cicuéndez^1,3^, Blanca González ^1,2,4^, Isabel Izquierdo-Barba ^1,2,4^*, Daniel Arcos ^1,2,4^*

1. Dpto Química en Ciencias Farmacéuticas, Facultad de Farmacia, Universidad Complutense de Madrid, Plaza Ramón y Cajal s/n, 28040 Madrid, Spain

2. CIBER de Bioingeniería, Biomateriales y Nanomedicina, CIBER-BBN, 28040 Madrid, Spain

3. Instituto de Investigación Sanitaria del Hospital Clínico San Carlos (IdISSC), Madrid, Spain

4. Instituto de Investigación Sanitaria, Hospital 12 de Octubre i+12, Madrid, Spain

Movie S1:

<https://drive.google.com/file/d/1NVUItP3lOt0rYRSck_diInLcBX-1L0Xh/view?usp=drive_link>

Movie S2:

<https://drive.google.com/file/d/19KxDREIEZiLtsWCubM1eftHs2UfpCz5P/view?usp=drive_link>

**Figure S1.** Thermogravimetric analysis of the different synthesized nanoparticles.

**Figure S2.** Nitrogen adsorption-desorption isotherm linear plots of the synthetized nanoparticles. A) MBGN; B) MBGN-L and C) MBGN-L-NAC isotherm linear plots.

**Figure S3.** Bioactivity of MBGN in contact with SBF. A) FTIR of the MBGN in contact with SBF for 0, 1 hour, 2 hours, 6 hours, 24 hours, 3 days and 7 days. B) TEM micrographs of MBGN in contact with SBF for 0, 3 and 7 days. C) EDX spectra of MBGN in contact with SBF for 0, 3 and 7 days.


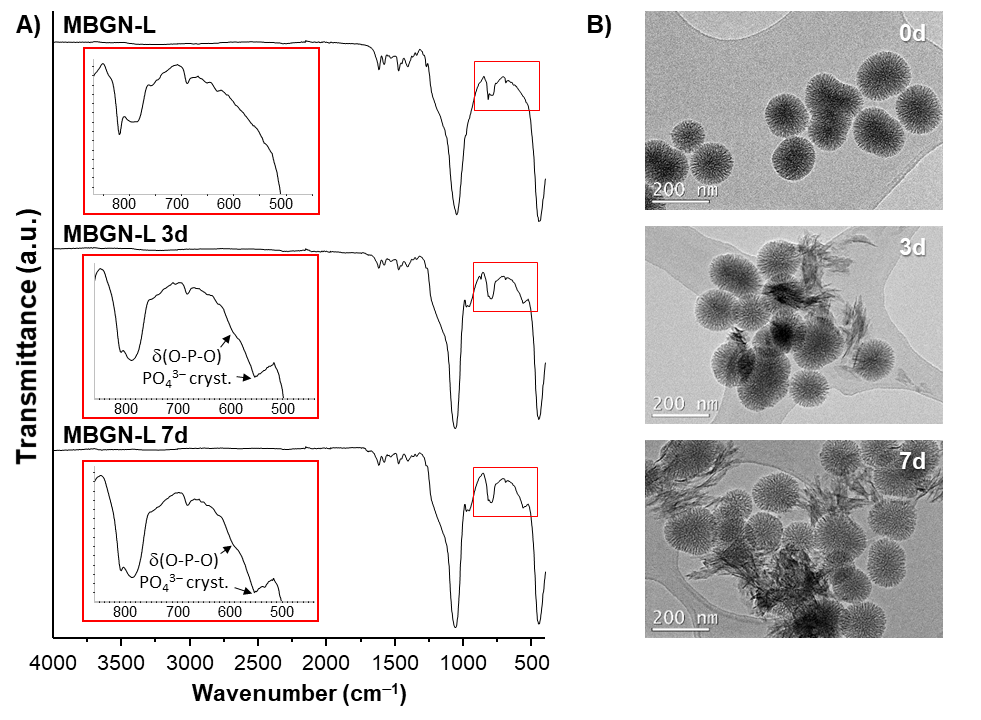


**Figure S4.** Bioactivity of MBGN-L in contact with SBF. A) FTIR of the MBGN-L in contact with SBF for 0, 3 and 7 days. B) TEM micrographs of MBGN-L in contact with SBF for 0, 3 and 7 days. C) EDX spectra of MBGN-L in contact with SBF for 0, 3 and 7 days.

**Figure S5.** FTIR spectra of MBGN-NAC (up) and MBGN-L-NAC soaked in SBF for 7 days (down).


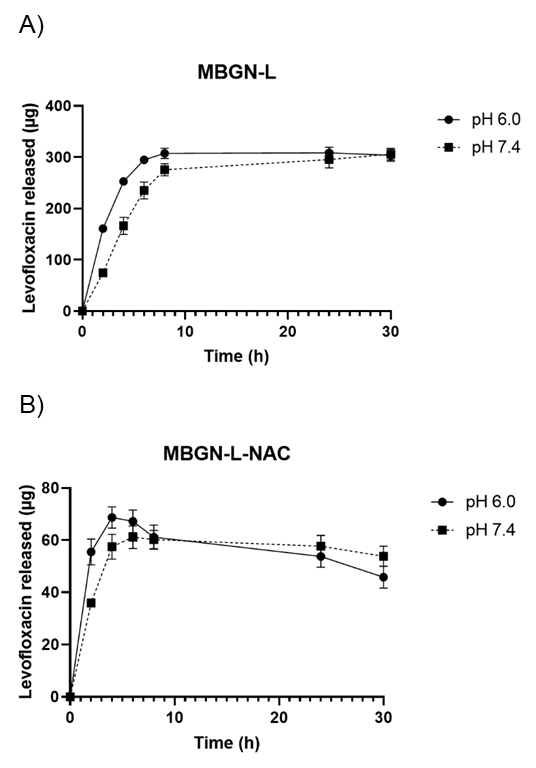


**Figure S6.** Comparative levofloxacin release between pH 6.0 and pH 7.4 in A) MBGN-L and B) MBGN-L-NAC
